# The Need to Invest in a Public Health System for Older Adults and Longer Lives, Fit for the Next Pandemic

**DOI:** 10.3389/fpubh.2021.682949

**Published:** 2021-06-17

**Authors:** Linda P. Fried

**Affiliations:** Columbia University Mailman School of Public Health, New York, NY, United States

**Keywords:** loneliness, COVID-19, isolation, aging, prevention, public health system

## Introduction

In Camus' *The Plague*, written in 1947, the plague comes to a fictitious city, Oran, on the Algerian coast. The authorities' response to the plague resonates with approaches to coronavirus 2019 (COVID-19): they isolate the town from the outside, and within the town isolate the infected to prevent spread. *The Plague* presents the experiences, responses, and traumas of people in many life situations, including the trauma when authorities remove infected family members from the household and take them to the sequestered building for those infected on the margins of town. It describes the pain for parents when they are separated from their children. It follows the trajectory of several older men who are infected—and some who escape infection even while wishing for it. Camus points out the economic distress of those who are poor when scarce food becomes costly, compared with the layers of protection for those better connected and resourced. Notably, there is little focus on the older population and no attention to older women, and it spends little time on what it means for an older person to be locked away in their last years, separated from those they love and who love them.

The US Centers for Disease Control and Prevention was founded in 1946, and the public health responses in the fictional Oran reasonably represent global approaches to pandemic containment at that time. The public health responses to COVID-19 also have deep parallels with Oran, obviously including containing the spread of disease by isolating the infected, quarantining the exposed, secluding the vulnerable, and preventing travel. We have many additional advantages now: scientific advances and capabilities; effective public health expertise in pandemic prevention and response; a recognition that clear, transparent, and aligned leadership and communication is essential; and an understanding of the use of physical distancing to minimize risk of spread, which combined with better face coverings and sanitizing together constitute an effective “non-pharmacologic vaccine.” Now, we have a pharmacologic vaccine which adds essential protection. However, the fact remains that with regard to COVID-19, it appears we have not progressed as much as one would have hoped from Oran's prevention methods, with significant mismatches between longstanding approaches and the current realities of this 2020–2021 pandemic.

## A Twenty-First Century Pandemic Reveals the Need to Edit the Twentieth Century Public Health Playbook

Compared with the last real pandemic of 1918, as well as the situation of the late 1940s, our population risk profile has changed dramatically. The USA, and the world, have demographically and epidemiologically different populations now: we are substantially older and with high rates of chronic diseases and obesity (Table 1). In this pandemic, those with obesity, multimorbidity, disability, and frailty, in particular, have shown high vulnerability to severe illness and death from COVID-19. In addition, while the 1918 Spanish flu pandemic found young adults—especially those in military barracks—most vulnerable, the most vulnerable in 2020–2021 have been older adults, medical care providers, those in close quarters in nursing homes–residents or staff—or prisons or meatpacking plants, and those with pre-existing health conditions at any age. People of color, both older and middle aged, have been particularly at risk due to the cumulative effects of systemic disadvantage, racism, and disparities. The USA's responses to containing the pandemic itself have not been as successful as they could be, because of lack of empowerment of science-based public health leadership and a public health system long starved of resources, workforce, and political support of its import (1), exacerbated by a primary focus on mitigating medical care needs without adequate prevention. We have not been prepared to target protection to people with pre-existing chronic disease, in congregate living situations, and other risk groups. Furthermore, current approaches have resulted in extreme social isolation for many older people; the latter, in particular, is inhumane and requires new approaches.

**Table 1:** The transitions in US demographic and population health status between two pandemics.

	**1920**	**2018**
Life expectancy at birth	48.4 years (males)	76.2 years (males)
	51.8 years (females)	81.2 years (females)
Population aged 65+	7.3%	16%
Population aged 85+	0.1%	1.9%
% of non-institutionalized 65+ years reporting fair or poor health	Unknown	21.7% (2017)
% of adults aged 65+ living with 2 or more chronic conditions	Unknown	63.7%

*Sources: Life expectancy at birth (1920) (2); Life expectancy at birth (2018) (3); Population percentages (1920) (4); Population percentages (2018) (5); Older adults reporting poor or fair health (2017) (6); Older adults reporting 2 or more chronic conditions (2018) (7)*.

A new commitment is needed to resolve the mismatch between existing capabilities and our population's realities and needs. This necessitates, at the core, investing in the actions which we must take collectively to protect the health of the population, the National Academy of Medicine definition of public health (8), with consistent, transparent, and evidence-based leadership from the national level and with alignment and synergy at the federal, state, and local levels, functioning as an integrated system. Expert public health leadership and partnership with communities in an adequately resourced public health system matching twenty-first century needs and capabilities requires building for a sustained and long-term view and—right now—preparing more aligned, effective, and humane approaches for use in the next pandemic and the creation of a healthier population less vulnerable in a pandemic.

To be effective for our twenty-first century reality, we need to modernize public health approaches so as to prevent both infection and severe isolation and other unintended consequences of infection-only responses to COVID-19. The long-term underinvestment in public health has revealed needs for modernized and integrated public health-led disease surveillance, and a trained and adequate public health workforce who can mount rapid and sufficient responses of contact tracing and testing. We need to develop approaches that recognize the most vulnerable and meet their needs in order to decrease risks of both infection and precarity. The pandemic has shown us threats to well-being at every level of Maslow's hierarchy of essential human needs (9), and particularly so for older adults and those with the cumulative effects of health disparities and with few resources, including precariousness of access to food, shelter, security, employment, and other resources. Furthermore, those at high risk are often living in multigenerational households with no available place to self-isolate. Additionally, the human need for connection has been severely threatened. Fifty-six percent of older adults have felt isolated from others, and 46% had infrequent social contact, twice the level of 2 years previously. Overall, rates of loneliness have been quite high in the pandemic, and especially so for those who are essentially living in a prolonged state of solitary confinement, whether homebound or in an institution. Those older adults experiencing loneliness are more likely to be women, to be living alone, unemployed, disabled or not working, or with incomes <$60,000 a year (10). Loneliness risk in this pandemic appears to be compounded by a pre-existing thinning, over the last half century, of social infrastructure and institutions that support social capital and positive connection and cohesion in a community (11). A sense of being a visible and valued member of a community—belonging, self-esteem, and self-actualization in the language of Maslow—has been further threatened by the pandemic-related rise in ageism, with public narratives that have both blamed older people for the severe impact of the pandemic and further devalued older people by proposing that they remain in seclusion so “productive” individuals (per the narrative) can get back to work. Little is said about the evidence that health and the economy are not separable, nor about the human rights and value of older people, or even to value older adults' accepting prolonged isolation in order to protect not just themselves from getting infected, but, altruistically, to keep others safe.

The impact of pandemic-induced social isolation has been felt across generations, with people of all ages reporting feeling a broad range of emotional distress—including fears regarding illness and death, economic survival, isolation and loneliness, loss of sense of control, hopelessness, and profound sadness (12). This is compounded by intergenerational grief at not being together. The solutions going forward need to include new methods to support both protection and connection in a pandemic. At the same time, we need to invest in the long game and rebuild society's social infrastructure and built environment to match our twenty-first century context, supporting thriving in communities, intra- and intergenerational connection and cohesion, and resilience to emergencies (11). As is always the case, we find that designs that benefit older people improve the lived experience for all ages.

The long view also demands a public health system able to deliver the programs through which we invest in preventing both chronic and infectious diseases and promoting health and resilience across the life course in every community. It is estimated that half of all chronic diseases are preventable; a public health system that has the resources and workforce to deliver this to all communities is needed. This will result in lowering the underlying population's vulnerabilities to infectious disease. By fully utilizing our knowledge about how to create population health, we will decrease the health disparities that result in vulnerability to infection, lower risks from future pandemics and lay the basis for healthy longevity.

A pandemic makes evident to all that public health is a public good, that collective actions are essential to contain a pandemic and protect health of all, with all benefiting and none excluded. The key will be to maintain this current realization and reinvest in what it takes to create a healthy population and sustain this. By definition, public goods require governmental investment since there is no profit to be made by any sector, while all benefit and none are excluded. New population vulnerabilities—compared with the pandemic of 1918—and decades of government disinvestment have rendered our public health system insufficiently able to respond to the crisis, and reveal (13) policies and programs ill-matched to the current needs of our society of longer lives, and with high rates of chronic conditions and health disparities. We need to now recognize these mismatches and develop a forward-looking twenty-first century public health system that has the capability to deliver sustainable approaches that create health resilience against future threats and prevent the development of ill health broadly. The areas for public health system leadership and programs to protect older adults and other vulnerable populations in the next pandemic can be framed as a public health system for all ages (14) that would include:

Capabilities for acute responses

(1) Next-generation public health programs and systems, aligned and integrated across federal, state, and local levels, that can agilely identify high risk groups and persons in a pandemic, and implement practices that both minimize infection risk and other coexisting threats to well-being, including precarity, decreased ability to obtain medical care for other conditions, enforced and prolonged social isolation and loneliness and loss of connection to others and sense of belonging (11, 15).
(2) Local public health departments, as community health strategists, need to promote and create policies that provide social protections during emergencies (i.e., eviction protections, access to food) in collaboration with other governmental entities (14). We should start by seizing this moment to do the research to understand where older people and all at risk were well-served, and the needs that should be met.
(3) The suspension of many community-based services left vulnerable older adults without food or meal deliveries, aides to take them to medical appointments, and visitors to bring services and counter loneliness and isolation. In the absence of these, precarity has risen, while other health problems have not been attended to. Working jointly with local agencies on aging and other community groups, public health departments should develop infection prevention regulations and guidelines for community organizations so that they can safely continue to care for and support homebound older adults, while also enabling continued in-home care by family members.
(4) Multigenerational households with few resources are at high risk for infection in a pandemic and require targeted approaches to offer quarantine to those exposed and isolation and care to those infected outside of their household, while supporting connection to loved ones.
(5) Furthermore, we need to develop population-level and community-based programmatic approaches to prevent the solitary confinement and loneliness arising from a pandemic with both acute responses and guidelines and long-term sustainable approaches (16, 17). This includes work, as led by the Hartford Foundation, to promote transition to models of smaller units of community-based long-term care, and smaller “pods” that would better prevent infection of residents and staff while maintaining interaction during the next pandemic (16). We could also develop roles, especially online, for community-dwelling older adults to support the well-being of their community during a pandemic or other emergency. These would counter the inaccurate ageist tropes that devalue older adults, while enabling solutions for social belonging, purpose, and impact for older people, essential to well-being. Roles could range from community education about how to protect oneself and each other from infection, to identifying neighbors in need, to community service of organizing food or meals for those in the building or neighborhood unable to get it, to online reading groups for younger children or online choral groups, and much more. This would require assuring Internet connectivity for all (see below).

These evolved approaches will provide the bases for resilience in the face of future pandemics and many other potential emergencies, such as weather-related events.

Long-term sustainable solutions:

(1) Prevention of chronic disease, disability, and frailty: The pandemic has revealed the differences in pandemic risk based on underlying ill health and the cumulative effects of health disparities, and the physiologic vulnerabilities of aging. Investment in local health departments' ability to deliver health promotion and disease prevention across the full life course and for the long term is crucial, so that the US population becomes healthier overall, and more resilient to health threats such as pandemics.
(2) Redesign of congregate living settings, including nursing homes, to enable connection as well as protection, as above.
(3) Finally, our community infrastructure of connection and cohesion needs to be strengthened and remodeled to protect against loneliness, with special attention to strengthening social capital and building this essential bulwark for a more resilient, thriving society. One essential approach involves designing the built environment in ways that foster connection. Another is to scale models for volunteerism by older adults in roles that strengthen communities and enable the dimensions of well-being that involve meaning and purpose in our longer lives (11). A third approach would be to recognize the critical import of Internet connectivity in today's world, and that many older adults live without that vital connectivity. Despite Internet access's centrality to life in the USA, the FCC's 2019 Broadband Deployment report noted that 21.3 million people in the USA lack access to broadband Internet; outside researchers estimate the number to be closer to 42 million (18). This is the time to make connectivity a public utility—a move that would benefit not just all older people and prepare us for future pandemics, but benefit as well the countless children, families, and adults of all ages who have found Internet access to be critical for education, jobs, and social connections.

## Conclusion

The COVID-19 pandemic has demonstrated that for all of our advances, our pandemic response has not effectively deployed our scientific knowledge and needs to be transformed to match our current twenty-first century realities and capabilities. The public health system needs to be updated to work as an integrated system at adequate scale. This will require substantial public investment to modernize and deliver health promotion and protection to all Americans, of all ages. Effective approaches for containing a pandemic require ability to quickly identify those at risk and have a repertoire of interventions tailored to different risk groups, including older adults. The approaches above would fit within the developing federal and state-level agendas to create an age-friendly public health system, would require federal leadership and state and local implementation, would be a basis for building a healthy population across longer lives, and would support many who are similarly vulnerable whether or not old.

## Author Contributions

The author confirms being the sole contributor of this work and has approved it for publication.

## Conflict of Interest

The author declares that the research was conducted in the absence of any commercial or financial relationships that could be construed as a potential conflict of interest.
